# Relationships among oncostatin M, insulin resistance, and chronic inflammation: a pilot study

**DOI:** 10.20945/2359-3997000000176

**Published:** 2019-09-25

**Authors:** Murat Akarsu, Mehmet Hurşitoğlu, Zeki Toprak, Şengül Aydin Yoldemir, Özgür Altun, Ilkim Deniz Toprak, Mustafa Özcan, Gülden Yürüyen, Bilal Uğurlukişi, Mahmut Genco Erdem, Kerem Kirna, Pinar Demir, Gazi Çapar, Yücel Arman, Tufan Tükek

**Affiliations:** 1 Okmeydani Training and Research Hospital Department of Internal Medicine Istanbul Turkey Okmeydani Training and Research Hospital, Department of Internal Medicine, Istanbul, Turkey; 2 Dr. Sadi Konuk Training and Research Hospital Department of Internal Medicine Istanbul Turkey Dr. Sadi Konuk Training and Research Hospital, Department of Internal Medicine, Istanbul, Turkey; 3 Gaziosmanpaşa Taksim Training and Research Hospital Department of Internal Medicine Istanbul Turkey Gaziosmanpaşa Taksim Training and Research Hospital, Department of Internal Medicine, Istanbul, Turkey; 4 Fatih Sultan Mehmet Training and Research Hospital Department of Internal Medicine Istanbul Turkey Fatih Sultan Mehmet Training and Research Hospital, Department of Internal Medicine, Istanbul, Turkey; 5 Şişli Etfal Training and Research Hospital Department of Internal Medicine Istanbul Turkey Şişli Etfal Training and Research Hospital, Department of Internal Medicine, Istanbul, Turkey; 6 Istinye Üniversity Medical Park Hospital Department of Internal Medicine Istanbul Turkey Istinye Üniversity, Medical Park Hospital Department of Internal Medicine, Istanbul; 7 Haseki Training and Research Hospital Department of Internal Medicine Istanbul Turkey Haseki Training and Research Hospital, Department of Internal Medicine, Istanbul, Turkey; 8 Istanbul University Medical Faculty Department of Internal Medicine Istanbul Turkey Istanbul University, Medical Faculty, Department of Internal Medicine, Istanbul, Turkey

**Keywords:** Diabetes mellitus, insulin resistance, oncostatin M, metabolic syndrome

## Abstract

**Objective:**

Activated macrophages (M1-type macrophages) in adipose tissue secrete many proinflammatory cytokines that induce insulin resistance (IR). Oncostatin M (OSM), a member of the interleukin-6 (IL-6) family of Gp130 cytokines, plays an important role in a variety of biological functions, including the regulation of inflammatory responses. Proinflammatory cytokines released in patients with IR trigger a chronic, low-grade inflammatory reaction in blood vessel walls. This inflammator response leads to endothelial damage, which is the main mechanism for atherosclerosis and many cardiovascular diseases. Animal studies have reported a relationship between OSM and IR. To the best of our knowledge, however, few clinical studies have examined this topic. Therefore, we studied the relationship between serum levels of OSM and IR.

**Subjects and methods:**

This prospective cross-sectional case-control study enrolled 50 people with IR (according to the HOMA-IR and QUICKI indices) and 34 healthy controls. The fasting blood concentrations of insulin, glucose, blood urea nitrogen (BUN), creatinine, aspartate aminotransferase (AST), alanine aminotransferase (ALT), low-density lipoprotein cholesterol (LDL-C), high-density lipoprotein cholesterol (HDL-C), triglyceride, total cholesterol, C-reactive protein (CRP), and OSM were determined.

**Results:**

There were no significant differences between the two groups in age, sex, and HbA1c levels. Univariate analyses showed that waist circumference (WC) and levels of fasting glucose, insulin, CRP, HDL-C, OSM, HOMA-IR, and QUICKI differed between the two study groups. In multivariate analyses, both IR indices (QUICKI and HOMA) and OSM differed between the two groups.

**Conclusion:**

OSM was correlated with the IR indices (QUICKI and HOMA). For simplicity, it might replace the other IR indices in the future. Further detailed studies are needed to confirm this.

## INTRODUCTION

Metabolic syndrome (MS) is an important health problem. It is a multi-factorial disorder influenced by interactions between genetic and environmental factors. Obesity and insulin resistance (IR) are closely associated with a state of chronic inflammation (1). Proinflammatory cytokines released in patients with IR trigger chronic, low-grade inflammation in blood vessel walls (2,3). The number of adipose tissue macrophages (ATMs) is higher in obese individuals and they participate in inflammatory pathways after activation in the adipose tissues. ATMs are the primary sources of proinflammatory and prothrombotic molecules in adipose tissue. Consequently, IR deepens in these tissues (4,5).

Hyperglycaemia triggers arterial inflammation via an increase in the production of advanced glycation end products. IR makes a significant contribution to atherosclerosis, even before diabetes develops (3).

Oncostatin is a member of the Gp130 cytokine family. By activating the JAK/STAT pathways, it affects cell growth, neuron development, the inflammatory response, and other physiological process (6,7). OSM signals via the LIF receptor-alfa (LIFR) and OSM receptor (OSMR) (8). OSM and the OSMR are potential therapeutic targets in chronic inflammatory diseases that cause endothelial dysfunction, such as rheumatoid arthritis, atherosclerosis, thrombosis, and abnormal angiogenesis (9-12).

OSM signalling may be required for homeostasis in both the liver and adipose tissue. The absence of OSM signalling causes obesity, hepatic steatosis, and IR (13). Although the relationship between OSM and IR has clearly been demonstrated in animal experiments, few clinical trials in humans have been conducted. Therefore, this clinical study examined the relationship between serum levels of OSM and IR.

## SUBJECTS AND METHODS

This prospective cross-sectional study was approved by our hospital’s ethics committee (protocol no. 731, dated 10/10/2017). The study enrolled 81 participants with a diagnosis of treatment-naïve IR. After a thorough evaluation, 30 were excluded based on the below-mentioned exclusion criteria. The remaining 51 comprised the IR group. The healthy controls were 33 sex- and age-matched non-IR individuals. Informed consent was obtained from all participants. All participants’ detailed medical and socio-cultural history and physical examination findings were recorded, including vital signs, weight, height, body mass index (BMI), and waist circumference (WC). After an overnight 8 h fast, a venous blood sample was obtained from each participant and analysed using standard procedures.

IR was evaluated using the homeostasis model assessment of insulin resistance (HOMA-IR) and quantitative insulin sensitivity check index (QUICKI). The two indices are calculated as follows: HOMA-IR = insulin (μU/mL) × Glucose (mg/dL)/405 QUICKI = (1/log insulin + log glucose) (mg/dL). Because there is no recommended threshold for IR in children, adolescents, or adults, a cut-off of 2.5 was used to separate participants into normal or elevated HOMA-IR groups (14). A QUICKI < 0.339 indicates IR. Both HOMA-IR and QUICKI compensate for fasting hyperglycaemia, and the results of the indices correlate reasonably well with those using the euglycaemic clamp technique (15).

Exclusion criteria were as follows: 1) a psychiatric or chronic illness (e.g., hypertension, diabetes mellitus, chronic renal failure, congestive heart disease, hyperlipidaemia, chronic obstructive pulmonary disease); 2) using drugs (antioxidants, vitamins, antibiotics, oral antidiabetics, antihypertensive, and psychiatric and other drugs); 3) younger than 18 years or older than 80 years; 4) any inflammatory disease or medication that could potentially interfere with the measurement of OSM; and 5) inability to give informed consent.

### Measurements

BMI was calculated as weight (kg)/height^2^ (m^2^). Obesity was defined as BMI > 30 kg/m^2^. WC was measured by placing a tape in the horizontal plane at the level of the iliac crest. Following an 8 h fast, blood samples were collected from all participants at 07.00–08.00 AM and stored at –80°C until testing. Serum levels of cholesterol, triglycerides, high-density lipoprotein cholesterol (HDL-C), glucose, creatinine, and urea were measured via enzymatic colorimetric methods using commercial kits (COBAS-8000, Roche Diagnostics, Mannheim, Germany), and low-density lipoprotein cholesterol (LDL-C) was calculated using the Friedewald formula.

Blood levels of HbA1c were determined via high-performance liquid chromatography usingr a Premier Hb9210 kit (Trinity Biotech). Final results were expressed as percent HbA1c of the total haemoglobin according to the protocol of the Diabetes Control and Complications Trial/National Glycohaemoglobin Standardisation Program (DCCT/NGSP). The insulin level was determined through an electrochemiluminescence immunoassay using a COBAS-8000 kit (Roche Diagnostics, Mannheim, Germany). Serum levels of C-reactive protein (CRP) were quantified using an enzyme-linked immunosorbent assay (ELISA).

### OSM measurements

A Human Oncostatin M kit (catalogue number: E-EL-H2247 by ELISA) was used for the measurements. The linear analytical detection range was 1 to 1000 pg/mL. The minimum detection limit was 0.14 pg/mL and the sensitivity was 1 pg/mL. The lower limit of detection was determined by assaying replicates of zero and the standard curve. To estimate OSM levels, 10 mL peripheral venous blood was put in plain blood collection tubes with no additives and immediately immersed in ice and allowed to clot for 1 h before centrifugation (2000 *g* at 4°C for 15 min). The serum was stored at –80°C until analysed. The samples were thawed and OSM levels were measured using ELIZA (Farmasina Lab, Okmeydani, Istanbul, Turkey).

### Statistical analyses

SPSS 22.0 (IBM Corporation, CA, USA) was used for the statistical analyses. Normally distributed data are expressed as the mean ± SE. Otherwise, the median and range are given. Independent quantitative measures were analysed using the independent samples *t*-test and Mann–Whitney *U*-test. Independent qualitative data were analysed using the chi-square test. A two-sided *P* < 0.05 was considered significant.

## RESULTS

**Figure 1:**
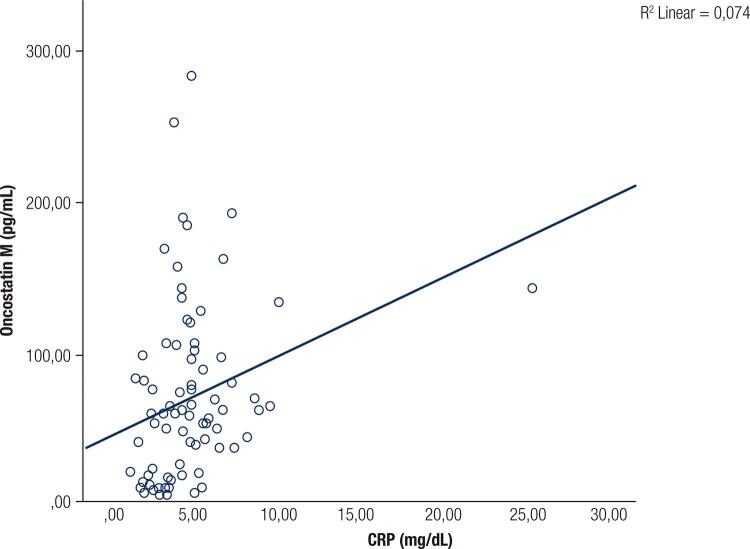
Relation between serum oncostatin M by CRP.

There were no significant differences in descriptive statistics between the two groups in terms of age, sex, HbA1c, blood urea nitrogen (BUN), creatinine, aspartate aminotransferase (AST), alanine aminotransferase (ALT), LDL-C, triglyceride, and total cholesterol. BMI, WC, glucose, insulin, HOMA-IR, QUICKI, CRP, HDL-C, and OSM differed significantly between the two groups in univariate analyses (Table 1). In multivariate analyses, only the IR indices (QUICKI and HOMA) and OSM differed between the groups (Table 2). There was a strong correlation between OSM and the inflammation marker CRP (Figure 1).

**Table 1:** IR and non-IR group laboratory features

	Control Group (non-IR)		Case Group (IR)		p
	
	Mean ± s.d /n%	Median	Mean ± s.d /n%	Median	
Age	42,9 ± 11,0	43,0	47,7 ± 11,4	47,0	0,064^t^
Gender					
Male	6	18,2%	15	29,4%	0,246^X^2^^
Female	27	81,%	36	70,6%	
Weight (kg)	79,1 ± 15,7	80,0	86,5 ± 10,9	85,0	0,055^t^
Length (cm)	161,8 ± 8,0	163,0	162,8 ± 7,6	162,0	0,542^t^
BMI (kg/m^2^)	30,4 ± 6,3	30,1	32,7 ± 4,3	32,3	0,064^t^
WAIST (cm)	82,4 ± 8,4	80,0	92,3 ± 8,8	92,0	**0,000**^m^
F. glucose (mg/dL)	87,5 ± 9,3	87,0	99,3 ± 12,5	101,0	**0,000**^m^
F. insulin (mg/dL)	7,7 ± 2,8	8,1	22,0 ± 12,2	20,2	**0,000**^m^
QUICKI index	0,4 ± 0,0	0,4	0,3 ± 0,0	0,3	**0,000**^m^
HOMA index	1,6 ± 0,5	1,8	5,4 ± 3,2	4,7	**0,000**^m^
HbA1C (%)	5,7 ± 0,4	5,6	5,7 ± 0,4	5,8	0,351^m^
OSM (pg/mL)	28,8 ± 27,7	15,0	93,7 ± 58,6	75,8	**0,000**^m^
CRP (mg/dL)	2,8 ± 0,9	2,5	5,8 ± 3,2	4,9	**0,000**^m^
BUN (mg/dL)	22,0 ± 6,6	22,0	24,1 ± 7,4	24,0	0,062^m^
Creatinine (mg/dL)	0,8 ± 0,5	0,7	2,4 ± 11,8	0,8	0,097^m^
T. Cholesterol (mg/dL)	189,9 ± 34,5	189,0	198,2 ± 47,3	185,0	0,677^m^
LDL (mg/dL)	108,8 ± 38,9	110,0	123,4 ± 43,0	120,0	0,276^m^
HDL (mg/dL)	50,5 ± 11,0	50,0	44,0 ± 10,6	42,0	**0,015**^m^
TG (mg/dL)	115,5 ± 56,2	102,0	170,8 ± 112,8	132,0	**0,011**^m^

^m^ Mann-whitney u test; ^t^ t test; ^X^2^^ Chi-square test.

**Table 2:** Univariative and multivariative analysis

	Univariate model			Multivariate Model		
	
	OR	% 95 CI	p	OR	% 95 CI	p
WAIST (cm)	1,145	1,073 - 1,223	**0,000**			
F. Glucose (mg/dL)	1,106	1,050 - 1,165	**0,000**			
F. Insulin (mg/dL)	2,630	1,555 - 4,448	**0,000**			
QUICKI index	0,000	0,000 - 0,000	**0,000**	0,000	0,000 - 0,000	**0,006**
HOMA index	1,6	1,3 - 1,9	**0,000**	1,054	1,152 - 1,741	**0,036**
OSM (pg/mL)	1,046	1,025 - 1,068	**0,000**	1,072	1,002 - 1,147	**0,043**
CRP (mg/dL)	8,746	3,392 - 22,549	**0,000**			
HDL (mg/dL)	0,946	0,905 - 0,988	**0,012**			
TG (mg/dL)	1,009	1,002 - 1,016	**0,016**			

Logistic regression analysis.

## DISCUSSION

**Figure 2:**
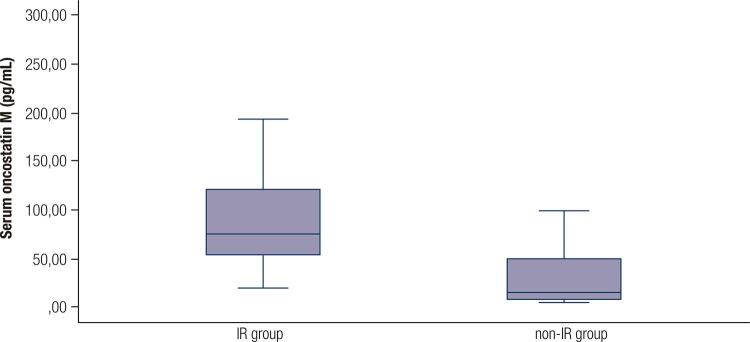
OSM levels in IR (case group) and non-IR (control group).

**Figure 3:**
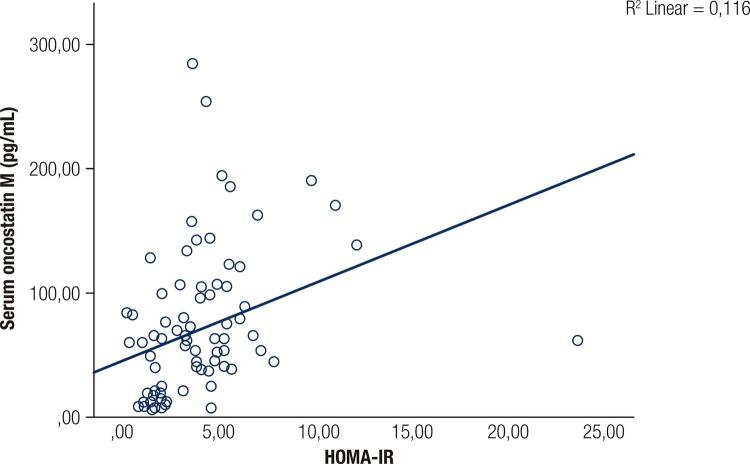
Relation between serum oncostatin M with HOMA-IR.

OSM is a member of IL-6 family of cytokines. In obese individuals, it is associated with increased levels of macrophages in adipose tissue and IR (16,17). In obese mice, OSM is produced by pro-inflammatory cytokines such as TNF-α. It is secreted by M1 adipose tissue macrophages (ATMs) and inhibits insulin-activated glucose transport to the tissues, particularly skeletal muscles, inducing IR (18,19). M2 ATMs secrete the anti-inflammatory cytokine IL-10. A change in the M1/M2 ratio of ATMs in adipose tissue leads to a change in the pro-inflammatory/anti-inflammatory cytokine ratio and the development of IR. Animal studies have reported an association between OSM and IR and have suggested that it is a novel therapeutic target for metabolic syndrome (6). We also found a close relationship between OSM and IR indices (Tables 1 and 2, Figures 2 and 3).

Many methods are used to assess IR in basic and clinical studies, including the insulin tolerance test, hyperinsulinaemic euglycaemic clamp test, HOMA-IR, QUICKI, and continuous infusion of glucose with model assessment (CIGMA) (20,21). We evaluated IR using the widely used HOMA-IR and QUICKI formulas. Univariate analyses indicated differences in HOMA-IR, QUICKI, WC, CRP, and OSM between the groups. In multivariate analyses, however, only IR indices and OSM differed significantly (Table 2). Some studies have suggested that QUICKI assesses IR better than HOMA-IR (22). In our study, the OSM level was correlated with both indices.

Could OSM replace these indices for the determination of IR? Further studies are needed to assess this. Although our sample size was small for determining a cut-off point, receiver operating characteristic analyses showed that a 30 ng/dL cut-off level for OSM predicted IR with a sensitivity of 94.1% and specificity of 69.7% (Table 3). Therefore, this single serum parameter might replace the multi-parametric IR estimation tests such as QUICKI or HOMA-IR for screening and diagnosing complicated IR-associated diseases such as metabolic syndrome and diabetes. Further detailed studies are needed to examine this.

**Table 3:** Sensitivity and specificity for OSM

	AUC	% 95 CI	p
Oncostatin M	0,864	0,783 - 0,945	**0,000**
Cut Off 30	0,819	0,716 - 0,922	**0,000**
		Sensitivity	94,1%
		Positive predictive value	82,8%
		Specificity	69,7%
		Negative predictive value	88,8%

Analysis with ROC curve.

In obesity, Timp1 (tissue inhibitor of metalloproteinases 1), Spp1 (osteopontin/secreted phosphoprotein 1), PAI-1 (plasminogen activator inhibitor-1) and Igfbp3 (insulin-like growth factor-binding protein 3) significantly increase in adipose tissue and contribute to the insulin resistance (23-27). OSM treatment regulates the expression of many proinflammatory adipokines causing insulin resistance (Timp1, PAI-1, Igfbp3, and Spp1) in adipose tissue (28). The ability of OSM to regulate the expression of these mediators, which cause insulin resistance, can give directions to insulin resistance monitoring and treatment. In these studies, the fact that OSM is in close relationship with proinflammatory cytokines in insulin resistance supports our study. In a study of pregnant women, it was shown that the serum OSM levels, and IL-6 and IL-1β expressions correlated with each other in relation to the inflammatory process (29). In another study by Li and cols. on patients with coronary artery disease, it was found that serum OSM and CRP levels were elevated in the patient group. Elevation of OSM levels in combination with CRP as an inflammatory marker was also consistent with our results (30).

One of the limitations of our study was that it was a cross-sectional observational study. We cannot determine whether improving the IR status (by lifestyle modification or medicines) would affect the OSM level.

Both obesity and IR are associated with chronic inflammation. In our study, WC was significantly higher in the IR group, while the BMI difference did not reach significance (Table 1). Central obesity (i.e., an increased WC) is a mandatory International Diabetes Federation diagnosis criterion for metabolic syndrome (i.e., IR associated status). BMI > 30 kg/m^2^ can replace WC for the diagnosis of this IR-related disease condition. This may explain the increased WC in the IR group. Another point supporting our interesting finding is the increased OSM levels in cachectic IR cancer patients (13). This close association between OSM levels and central obesity and IR makes OSM a potential marker in screening for central obesity and IR. Further detailed studies with larger sample sizes are needed to confirm these findings.

In conclusion, OSM is associated with IR and chronic inflammation. Our results indicate that OSM correlated with the IR indices HOMA-IR and QUICKI. Given its simplicity, OSM may replace these time-consuming IR-determining indices in the future. Further detailed studies need to examine this further.
